# The impact of music industry digitalisation on population well-being: Empirical evidence from China

**DOI:** 10.1371/journal.pone.0326247

**Published:** 2025-07-02

**Authors:** Chuanyi Liu, Yinghui Wang

**Affiliations:** The Graduate School Arts & Culture, Sangmyung University, Seoul, South Korea; The Open University of Israel, ISRAEL

## Abstract

With the profound transformations brought about by digital technology in the global economy and social operations, digital music has emerged as a vital medium for meeting residents’ cultural needs, playing a significant role in promoting cultural dissemination and catering to personalized cultural demands. Previous research has mainly focused on the commodity attributes, symbolic functions, and instrumental value of digital music at the micro level, while studies examining the potential impact of music industry digitalization on residents’ well-being remain relatively scarce. Addressing this gap, this study empirically investigates the impact and underlying mechanisms of music industry digitalization on residents’ happiness by constructing a fixed-effects model based on data from the 2022 China Family Panel Studies (CFPS). The results reveal that: (1) the digitalization of the music industry significantly enhances residents’ subjective well-being; (2) digitalization promotes happiness primarily by boosting social participation, increasing social trust, and improving mental health; and (3) compared to residents with higher Internet access frequency, higher educational attainment, and higher economic status, the positive effects of music industry digitalization on happiness are more pronounced among those with lower Internet access frequency, lower education levels, and lower economic status. This study provides new empirical evidence for understanding the social effects of digital cultural consumption and offers important insights for optimizing cultural policies and improving residents’ quality of life.

## 1. Introduction

Accompanied by the wave of digitization in the global context, the application of digital technologies—particularly the deep integration of Artificial Intelligence (AI)—has profoundly transformed the operational modes of the global economy and society. Digital music, as a key manifestation of the digital transformation of the music industry in recent years, has not only fundamentally changed the ways music is produced, disseminated, and consumed but has also gradually integrated into people’s daily lives, becoming an essential component of residents’ entertainment, leisure, and cultural consumption. According to the *China Digital Music Industry Report* (2023), the total output value of China’s digital music industry reached 190.75 billion yuan in 2023, representing a year-on-year increase of 22.7%. The booming development of the digital music industry indicates that digital music is increasingly becoming a major cultural consumption product for Chinese residents, playing a significant role in fulfilling their spiritual needs and promoting cultural dissemination.

The rapid growth of China’s digital music industry in recent years is closely linked to the development of the digital economy and changes in residents’ consumption patterns. On one hand, the widespread adoption of the Internet and mobile technologies has significantly lowered the barriers to music consumption. In the traditional music market, music was mainly distributed through physical media, which was costly and inconvenient to access. In contrast, digital music, characterized by immediacy, convenience, and diversity, transcends the limitations of time and space, allowing more residents to enjoy music anytime and anywhere. Additionally, the COVID-19 pandemic, which began in 2020, impacted the traditional offline music market and directly accelerated the development of online-based digital music [1]. On the other hand, in recent years, as Chinese residents’ disposable incomes have increased and their consumption structures have optimized, the demand for cultural consumption has become richer and more personalized. From this perspective, digital music—with its more diversified forms compared to traditional music, such as online music, short music videos, and live streaming performances—better caters to residents’ personalized cultural needs.

Music today is more of a resource than a cultural commodity. People engage with digital music across different contextual environments, situations, and levels of involvement [2]. Koh, Hann [3] state that the trend of music digitization can be divided into two main phases. The first phase involves the sale of physical and digital music records, while the second phase is characterized by the rise of streaming, unbundling, and cross-platform services that integrate music with other forms of entertainment, such as games, films, and television productions [4]. Consequently, research on digital music is often significantly differentiated based on the context of its use. Specifically, existing studies have primarily examined digital music through the lenses of its commodity attributes, instrumental functions, and cultural symbolism..

Firstly, the commodity attributes of digital music necessitate the study of its distribution strategies and profitability. At the beginning of this century, oligopolies such as Sony, BMG, Warner, and others held an almost complete monopoly over the global physical music industry. The development of digital music has clearly broken this monopoly and significantly lowered barriers to entry in the music industry [5]. This has led to an interesting phenomenon where music producers and products marginalized in the traditional physical music market have been able to compete with more established artists through online distribution channels. Record labels have managed to turn a profit through large-scale, small-batch music productions [6]. This shift brought about by digital technology has been described as the “long tail effect” [7]. Brynjolfsson & Hu [8] further explored the long tail effect in the online marketplace, demonstrating that lower search costs on the Internet drive consumers to seek out previously inaccessible niche products, thereby boosting the marketability of such products from the demand side. Furthermore, Eiriz & Leite [9] conducted an empirical study on the online music market, confirming the existence of the long tail effect. Their findings suggest that the growth of digital music has opened up new business opportunities for marginalized niche musicians, while also prompting a shift in business models within the music market. The use of digital technology has compelled these musicians to transition from pure artists to entrepreneurs engaged in multiple business activities, encompassing music production, promotion, distribution, and live performances.Secondly, digital music possesses distinct instrumental attributes. Based on the “stimulus-organism-response (SOR)” theory, listeners’ cognitive and behavioral responses can be significantly influenced during their interaction with music, for example, enhancing efficiency and achieving therapeutic effects. A review of relevant studies shows that, according to the heterogeneous differences in digital music usage contexts and listenership, research can be roughly divided into studies on learning performance among minors and studies on work performance among young adults. On one hand, music listening can affect learning performance by relaxing the mind and body, reducing boredom, and improving concentration [10] as well as enhance job performance [11]. One of the most influential studies comes from McLachlan [12], who introduced the concept of the “Mozart effect,” finding that listening to ten minutes of Mozart’s *Sonata for Two Pianos in D Major* (K. 448) improved participants’ performance on a spatial reasoning task. Subsequently, a large body of research emerged exploring the effects of classical music on human cognitive performance [13–15]. In the field of cognitive neurology, studies have pointed out that listening to Mozart’s music can reduce listeners’ EEG activity and enhance cognitive performance [16], as well as increase alpha rhythm activity in both healthy older adults and children [17]. However, some empirical studies suggest that the Mozart effect may not be universally present across all populations. For example, Silva & Belim [18] found that the Mozart effect did not occur in younger individuals but was observed only among some older adults. On the other hand, digital music users often rely on various forms of online music services for different listening purposes [19]. Due to the convenience of online music, users can interact with musical works more promptly to achieve their intended outcomes more efficiently [20]. In addition, the long-tail effect allows users’ marginalized and niche music needs to be better satisfied.

Finally, music as a cultural symbol is directly linked to the musical identity of its listeners. Sinclair & Tinson [21] proposed the concept of psychological ownership of music, suggesting that consumers of certain types of music use psychological ownership as a way to express loyalty to a music label, project their social identity, and align themselves with their musical identity. Although the emergence of digital music has lowered the cost of accessing various types of music products, it has also affected users’ sense of psychological ownership. However, the expansion of users’ social networks has further shaped digital music identity labels and fulfilled the social needs of different music audiences through hobbies, communities, and other means. Moreover, online music platforms allow fans to communicate directly with musicians and even achieve self-fulfillment through interactive behaviors such as mutual following [22]. This has directly raised concerns regarding the relationship between digital music and personal well-being [2]. Some studies have explored the impact of music on individual well-being; for instance, Morinville & Miranda [23] conducted a study on a sample of 229 Canadian youths, noting that music listening directly enhanced the subjective well-being of these adolescents

Across the aforementioned music-related studies in the fields of economics, management, social psychology, cognitive neurology, and others, it is evident that the impact of the digital transformation of music has been extensively explored. However, most research has focused primarily on areas such as business models, product strategies, and user behavior, while the investigation of its broader social effects remains insufficient. Although a small number of studies have attempted to reveal the impact of music consumption on user happiness, these analyses are often based on traditional music or limited to a single region, neglecting the unique attributes of digital music as an emerging consumption model. Smaller and more concentrated sample sizes also often limit the generalizability of their findings. In reality, digital music is not only a consumer product but also an important social tool and information carrier, whose impact on user well-being extends beyond direct listeners by influencing non-direct listeners through social networks, background music [24], and other channels. This highlights the necessity of studying how digital music affects the general well-being of the population. However, existing studies have yet to provide a detailed answer to this question. Therefore, by utilizing the China Family Panel Studies (CFPS), a large-scale micro-survey database from China, this study aims to explore whether the development of digital music influences the overall happiness of the Chinese population, leveraging a larger sample size and highly heterogeneous sample characteristics.

Overall, this study aims to address the following research questions: (1) Does the digitalization of the music industry significantly enhance residents’ subjective well-being? (2) Through which mechanisms does music industry digitalization influence residents’ well-being? (3) Does this impact exhibit heterogeneity across groups with different levels of Internet usage frequency, educational attainment, and economic status? By answering these questions, this study not only broadens the research perspective on the social effects of digital cultural consumption but also provides empirical evidence and practical insights for the formulation of cultural policies and the narrowing of socio-cultural disparities..

In the remainder of this study, we construct the theoretical framework and elaborate on it in detail in Chapter Two. Chapter Three focuses on the empirical strategy adopted for the study. Chapter Four presents the empirical results, while Chapter Five provides the conclusions, policy recommendations, and directions for future research..

## 2. Theoretical framework and hypothesis development

There is no doubt that the factors influencing residents’ subjective well-being are complex and multifaceted. Drawing on the SOR theory and integrating existing research on the commodity, instrumental, and symbolic functions of digital music, this study focuses on three mechanism pathways—social participation, social trust, and mental health—to systematically explore how the digitalization of the music industry affects residents’ well-being.

### 2.1. Social participation

The commodity attributes of digital music have socialized the mass music consumption process, making residents’ social participation an important component of digital music usage. Social participation reflects an individual’s social integration and sense of civic responsibility, primarily referring to the degree and manner of an individual’s engagement in social activities. Online music platforms (e.g., Spotify, NetEase Cloud Music) have significantly amplified the social attributes of music, transforming audience interaction from merely consumption based on personal preferences into a form of social participation. These platforms create opportunities for users to share, interact, and experience music collectively through built-in social modules, thereby facilitating connections among users through digital music. More importantly, extensive personalized recommendations and social interaction features provide users with virtual communities based on shared interests and preferences, where individuals can find a sense of belonging and identity [25].

Most directly, the sharing functions provided by digital music platforms greatly facilitate individual social interaction and participation. By sharing songs, albums, and playlists, users are not only able to express their musical tastes and emotions but also to share their personal experiences and emotional worlds with others. This act of sharing music offers users opportunities to interact with friends, family, colleagues, and even strangers, making music a bridge for interpersonal communication. Through these interactions, users’ sense of social engagement continues to grow as they build emotional connections based on their shared love of music, further expanding and deepening their social networks [26].

In addition, the development of digital music has broadened the scope of individuals’ participation in social activities. Through digital music platforms, users can engage in activities such as online concerts and live-streamed performances, which not only provide entertainment experiences but also create opportunities for shared participation. For instance, Vocaloid music—which utilizes artificial intelligence technology to create human-like vocal tracks (e.g., Hatsune Miku) through virtual voice libraries—has emerged in recent years. These native digital-age musical works are disseminated via digital music platforms, encouraging fans to use voice libraries for self-creation [27]. Users communicate about their creative experiences, share songs, and participate in live broadcasts of virtual singers through these platforms, thereby transitioning from passive listeners to active participants in music creation and community building. Through this interaction between music and listeners, individuals are able to shape and affirm their social identities and enhance their sense of social participation. Based on this, the following research hypothesis is proposed:

**Hypothesis H1**: The development of digital music enhances individual social engagement, thereby increasing overall population well-being.

### 2.2. Social trust

The cultural symbolism of digital music has gradually transformed it into an important medium for individuals to express themselves, construct their identities, and connect with society. On digital music platforms, users not only consume musical works but also express their aesthetic preferences and social identities through their affinity for specific genres, artists, lyrics, or emotional atmospheres. In this process, users with shared musical preferences form interest-based communities, and through activities such as sharing, commenting, and collecting, they continuously strengthen emotional bonds with one another. This sense of group belonging, centered around musical content, not only promotes interpersonal interactions but also invisibly contributes to the accumulation of social trust.

Social trust, a concept derived from psychology, primarily refers to individuals’ intuitive feelings toward others’ behavior, leading to approval or rejection. In the virtual digital era, where face-to-face communication is absent, users cannot easily assess others’ intentions and authenticity through facial expressions, tone, or contextual cues, thus exacerbating psychological distance and undermining social trust [28]. However, digital music offers a somewhat different scenario. Through emotional expression, music enables communication across geographical, cultural, and linguistic barriers, transforming what was traditionally a private experience into a collective and interactive one. Groups of people who appreciate the same songs or musical styles are more likely to create emotional resonance, which can aggregate into a collective sense of shared identity and externalize into a bond of trust through commenting and sharing on digital music platforms [29].

Moreover, the social trust cultivated through musical interactions extends into users’ real-life social behavior patterns, making it easier for individuals to build trust and intimacy in offline interactions, thereby enhancing their overall sense of social trust. In addition, digital music platforms provide spaces where users can express themselves and display their personalities and emotions, operating in both private and public dimensions. Whether through sharing playlists, collaborating on music creation, or interacting in comment sections and live broadcasts, individuals find value in their identities and maintain relationships through cooperative interactions. The foundation of these cooperative relationships is trust among strangers. For example, in the case of Vocaloid music, creating a complete piece—including voice library training, lyric writing, arrangement, and mixing—often requires collaborative efforts, as the learning costs are too high for individuals to bear alone. Cooperation within the community thus becomes a more efficient strategy, strengthening both social interaction and mutual trust. This trust-based digital music network not only boosts creators’ motivation and confidence, thereby improving their subjective well-being, but also enhances communication among fans, offering psychological support and promoting subjective well-being through co-constructed symbolic imagery.

Based on this, the following research hypothesis is proposed

**Hypothesis H2:** The development of digital music enhances individuals’ social trust, thereby increasing overall population well-being..

### 2.3. Mental health

The instrumental attributes of digital music suggest that it can serve as an important tool for intervening in individuals’ emotional states and alleviating psychological stress at any time. With the diverse scenarios provided by digital music platforms, individuals can select music resources that match their psychological conditions, thereby achieving self-regulation and emotional restoration. From this perspective, the connection between music and listeners’ physical and mental health is direct [30,31]. As digital technology has advanced, music has increasingly entered daily life as a vital tool to help people manage their emotions, relieve stress, and enhance their sense of well-being. On one hand, digital music can activate cognitive mechanisms related to embodied experience through its rhythm, melody, and emotional expression, thereby influencing individuals’ emotional regulation and psychological experiences [32] For many individuals, music serves as a powerful emotional support and a direct outlet for emotional catharsis [33], meaning that digital music can directly affect emotional responses and physiological states, helping to relieve anxiety, reduce depression, and even improve sleep quality [34]. On the other hand, by recording and sharing their music preferences, emotional states, and creative processes, individuals not only achieve emotional release and self-support but also gain recognition and encouragement from others. This ongoing emotional support and psychological satisfaction play a crucial role in enhancing individuals’ overall well-being.

Moreover, digital music enables people to express and release inner emotions through music creation and participation. In traditional social interactions, growing social pressures often make it difficult for individuals to fully express their emotions and thoughts. In contrast, digital music offers a relatively private and free channel for emotional expression, particularly for those who prefer to regulate emotions through creation and interpretation. Music creation thus becomes an effective method of self-distancing. For example, fans of the Vocaloid music genre can create and interpret songs through virtual singers, expressing their inner emotions via musical works. This form of creation not only serves as a medium for emotional expression but also promotes self-awareness and emotional self-regulation. Based on this, the following research hypothesis is proposed:

**Hypothesis H3: The development of digital music enhances individuals’ mental health, thereby increasing overall population well-being.**

**Fig 1:**
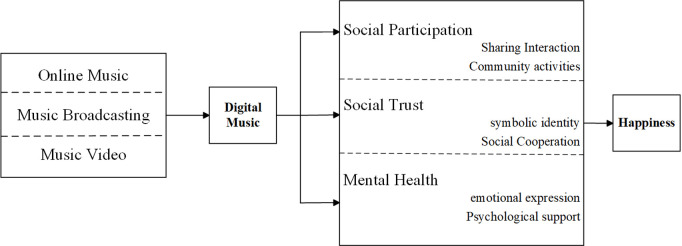
Theoretical framework.

Fig 1 presents the core logic of the hypothesis development in this paper.

## 3. Research design

### 3.1. Data and sample selection

The main data used in this study consist of two parts. The first part comes from the official song list information of QQ Music and NetEase Cloud Music. Using a Python crawler, we collected the official annual song summary data for 2022 from these two major online music platforms in China. The information gathered includes the song title, artist, release date, album, and the record label responsible for distribution. The second part of the data is drawn from the China Family Panel Studies (CFPS) database, a nationwide comprehensive social tracking survey project implemented by the China Centre for Social Science Survey at Peking University. Conducted biennially, the CFPS aims to capture changes in China’s social, economic, and health conditions by systematically collecting data from Chinese residents. The CFPS employs a multilevel, multistage stratified probability sampling method proportional to the population size, ensuring the representativeness of the sample. This study utilizes data from the sixth wave of the CFPS, conducted in 2022.

Since the two datasets differ in dimensionality, a matching process was conducted as follows. First, as our study focuses on the Chinese digital music industry, non-Chinese songs were excluded from the crawled data. Additionally, not all Chinese songs collected are pure digital music products; for example, some originate from television shows, films, or other entertainment media, where music distribution is primarily rooted in these broader forms and not purely digital in nature. Therefore, such songs were further excluded. Second, we identified the provinces where record companies are registered based on publicly available industrial and commercial information. We then clustered the number of highly disseminated digital songs in 2022 at the provincial level. Songs whose distributing record companies were registered outside the targeted provincial categories were excluded. Finally, the CFPS microdata provide geographic information through the PID code. Based on the PID code, we matched the provincial-level digital music data to the individual microdata level. After this matching process, the final sample consisted of 15,971 respondents from 31 provinces across China.

### 3.2. Variables definition and measurement

#### 3.2.1. Explained variables.

The explained variable in this study is the overall subjective well-being (Hap) of provincial residents. Subjective data, rather than objective data, were chosen to measure happiness because subjective well-being offers a more holistic assessment, encompassing individuals’ cognitive and emotional responses to various aspects of life. Compared to objective indicators, subjective measures capture psychological and emotional dimensions of well-being that are often inaccessible through objective data and help avoid the dimensional limitations associated with objective indicators [35]. Accordingly, we utilized the CFPS survey question “How happy do you feel?” to obtain the subjective well-being scores for the sample. Responses were recorded on a 10-point scale, with higher values indicating greater levels of happiness.

#### 3.2.2. Explanatory variables.

The main explanatory variable in this study is the level of digital music development in the region where each sample respondent resides. Since users primarily access digital music through online platforms, digital songs with higher dissemination popularity on these platforms are assumed to represent higher-quality music content. Therefore, this study uses the number of highly disseminated digital songs (Dig) released on the two major music platforms—QQ Music and NetEase Cloud Music—in each province during 2022 as the explanatory variable. In the empirical analysis, the natural logarithm of (Dig + 1) was used to account for potential heteroscedasticity and to smooth the distribution

#### 3.2.3. Control variables.

Since the sample for this study is derived from a micro-level survey, individual demographic characteristics are expected to influence subjective well-being. Therefore, the following control variables were included: age (Age), gender (Gender), education level (Edu), marital status (Mar), household registration status (Hukou), employment status (Occ), political affiliation (Par), family size (Num), and per capita household income (Inc). Considering that middle-aged individuals typically face greater social pressures than teenagers and older adults—and may thus be more susceptible to the effects of digital music on well-being—we additionally introduced the squared term of age (Age^2^) to control for the potential non-linear impact of age on subjective well-being.

#### 3.2.4. Intermediate variables.

In the theoretical analysis section, we proposed three potential mechanisms through which digital music may affect population well-being. For social participation, considering that Chinese society is often characterized as a “society of favors,” individual gift expenditures are frequently used as an important proxy for measuring social participation levels [36]. Therefore, we use the natural logarithm of annual gift expenditures (Gif), as recorded in the CFPS, to characterize individuals’ social participation. For social trust, we use the CFPS question “Trust in strangers” as a proxy, with the score (Tru) measured on a continuous scale from 0 to 10, where higher values indicate greater levels of trust in strangers. For mental health, we use the CESD8 score (Centre for Epidemiological Studies Depression Scale, CESD) disclosed in the CFPS as a proxy variable (Men), with higher scores indicating worse mental health status.

The definitions and measurement of the main variables are shown in Table 1, and the descriptive statistics are shown in Table 2.

**Table 1:** Description of variables.

Variable type	Variable name	Definition and measurement
Dependent Variable	Hap	Individual subjective well-being scores.
Independent Variable	Dig	Log of the number of digital music with a high level of dissemination heat.
Controls	Age	Age of the sample.
	Age2	Square of age of the sample.
	Gender	Gender of respondents, 1 = male, 0 = female.
	Edu	Educational level of respondents.
	Mar	Marital status of respondents, 1 = married, 0 = unmarried.
	Hukou	Respondent’s hukou status, 1 = urban hukou, 0 = rural hukou.
	Occ	Respondent’s work status, 1 = employed, 0 = unemployed.
	Par	Party status of respondents, 1 = Communist, 0 = others.
	Num	Number of persons in the respondent’s household.
	Inc	Annual per capita household income of respondents.
Mediating Variables	Renq	Log of annual gifts noted by respondents.
	Trust	Respondents’ trust in strangers.
	Mheal	Respondents’ mental health scores.

**Table 2:** Descriptive statistics.

	N	mean	sd	min	max
hap	15,967	7.473	2.029	0	10
lndm	15,971	0.576	0.990	0	3.664
age	15,971	47.20	15.45	10	97
age2	15,971	24.66	15.19	1	94.09
gender	15,971	0.504	0.500	0	1
edu	15,971	9.206	4.752	0	22
marital	15,971	0.808	0.394	0	1
hukou	15,971	0.701	0.458	0	1
job	15,971	1.199	0.434	0	2
party	15,971	0.0129	0.113	0	1
ful	15,971	3.988	1.987	1	16
ec	15,971	10.02	1.066	0	15.75
renq	15,971	7.142	2.595	0	12.21
trust	15,971	2.669	2.296	0	10
mheal	15,971	0.0786	0.0225	0.0313	0.125

### 3.3. Econometric model specification

In order to empirically test the relationship between the digitalisation of the music industry and the role of residents’ happiness, this paper first establishes a benchmark regression model as shown in equation (1), and its corresponding regression equation is as follows:


$$\begin{array}{c} \begin{array}{c} {Hap}_{i,t}=\alpha +\beta {Dig}_{i,t}+\sum_{j}\gamma_{j}{Controls}_{i,j,t}+\rho_{t}+\varepsilon_{t}\; \end{array} \end{array}$$ (1)

where the subscripts i,t represent the sample individuals and the province they live in, respectively. ${Hap}_{i,t}$ is the subjective well-being score of the individual, and ${Dig}_{i,t}$ is the number of digital music releases in province t where sample i is located. $\sum_{j}\gamma_{j}{Controls}_{i,j,t}$ is the set of control variables used in this study. In addition, this study further controls for province fixed effects$\rho_{t}$.$\varepsilon_{t}$ is the random perturbation term. Table 2 below discloses the descriptive statistics of the main variables of this study.

## 4. Empirical results and analyses

### 4.1. The impact of music industry digitalization on residents’ well-being

Table 3 presents the baseline estimation results for the impact of music industry digitalization on residents’ subjective well-being. Columns (1) and (2) report the baseline results without introducing fixed effects, before and after the inclusion of control variables, respectively. Columns (3) and (4) report the corresponding results after incorporating fixed effects, again before and after adding control variables.

**Table 3:** Benchmark regression results on the impact of music industry digitisation on residents’ happiness.

	(1)	(2)	(3)	(4)
	hap	hap	hap	hap
lndm	0.097^***^	0.077^***^	0.067^***^	0.057^***^
	(5.98)	(4.62)	(3.76)	(3.17)
age		−0.112^***^		−0.114^***^
		(−15.52)		(−15.84)
age2		0.122^***^		0.123^***^
		(16.82)		(17.04)
gender		0.008		0.018
		(0.23)		(0.55)
edu		0.008^*^		0.005
		(1.71)		(1.13)
marital		0.878^***^		0.869^***^
		(18.82)		(18.65)
hukou		−0.054		−0.035
		(−1.38)		(−0.90)
job		−0.038		−0.054
		(−0.92)		(−1.33)
party		0.344^**^		0.358^**^
		(2.44)		(2.55)
ful		0.038^***^		0.054^***^
		(4.42)		(6.21)
ec		0.117^***^		0.108^***^
		(6.84)		(6.31)
Constant	7.417^***^	7.678^***^	7.541^***^	7.898^***^
	(399.65)	(30.11)	(211.62)	(30.54)
Observations	15,967	15,967	15,967	15,967
R-squared	0.002	0.041	0.008	0.046
Region FE	NO	NO	YES	YES

Notes: t-statistics in parentheses,

*** p < 0.01,

** p < 0.05,

* p < 0.1

As shown in Table 3, regardless of whether fixed effects are included, the coefficient for the core explanatory variable—music industry digitalization (lndm)—remains significantly positive both before and after the inclusion of control variables. This indicates that the digital development of the music industry has a significant positive impact on residents’ happiness. Specifically, after controlling for both fixed effects and a full set of control variables, the coefficient of music industry digitalization reported in column (4) is 0.057 and is significant at the 1% level. This result suggests that, holding other factors constant, a 1% increase in the level of music industry digitalization in a given region is associated with an average increase of 0.057 units in residents’ subjective well-being.

### 4.2. Robustness tests

To verify the robustness of the main findings, this study conducts a series of robustness tests following the baseline regressions. Specifically, we perform four additional tests: replacing the baseline model with an ordered logit model, excluding extreme samples, addressing potential omitted variable bias, and excluding samples from the four major municipalities.

First, given that residents’ happiness is measured as a discrete ordered variable, we replace the baseline linear regression model with an ordered logit model for robustness testing. The corresponding results are presented in column (1) of Table 4.

**Table 4:** Robustness test results.

	(1)	(2)	(3)	(4)
	Replacement of ordered logit models	Removal of extreme samples	Adding control variables	Excluding the four largest municipalities
	hap	hap	hap	hap
lndm	0.028^**^	0.052^***^	0.079^***^	0.036^*^
	(1.97)	(2.77)	(4.71)	(1.77)
age	−0.104^***^	−0.117^***^	−0.114^***^	−0.116^***^
	(−15.81)	(−15.68)	(−15.47)	(−15.37)
age2	0.114^***^	0.126^***^	0.123^***^	0.125^***^
	(17.15)	(16.80)	(16.83)	(16.48)
gender	0.014	0.031	0.005	0.012
	(0.49)	(0.93)	(0.15)	(0.36)
edu	−0.002	0.004	0.009^*^	0.006
	(−0.43)	(0.87)	(1.94)	(1.27)
marital	0.753^***^	0.866^***^	0.875^***^	0.860^***^
	(17.81)	(17.98)	(18.75)	(17.62)
hukou	−0.043	−0.075^*^	−0.058	−0.024
	(−1.24)	(−1.85)	(−1.48)	(−0.57)
job	−0.039	−0.066	−0.040	−0.071^*^
	(−1.06)	(−1.58)	(−0.98)	(−1.67)
party	0.274^**^	0.406^***^	0.348^**^	0.340^**^
	(2.33)	(2.79)	(2.47)	(2.32)
ful	0.034^***^	0.051^***^	0.037^***^	0.055^***^
	(4.43)	(5.73)	(4.33)	(6.08)
ec	0.093^***^	0.102^***^	0.119^***^	0.114^***^
	(6.02)	(5.70)	(6.93)	(6.31)
xxjs			−0.000	
			(−1.26)	
Constant		8.105^***^	7.727^***^	7.880^***^
		(30.23)	(29.96)	(29.08)
Observations	15,967	14,825	15,967	15,079
R-squared		0.047	0.041	0.043
Region FE	YES	YES	YES	YES

Notes: t-statistics in parentheses,

*** p < 0.01,

** p < 0.05,

* p < 0.1.

Second, considering that the CFPS data are derived from survey responses, there may be subjective bias introduced by some respondents during the data collection process. To address this, we use the respondents’ “degree of eagerness to end the interview” recorded in the CFPS as an indicator and exclude those samples that exhibited a high eagerness to terminate the survey. The results after this exclusion are shown in column (2) of Table 4.

Third, recognizing that Internet usage frequency is closely related to residents’ well-being—since individuals access the positive externalities of the digital music industry primarily through the Internet—we introduce individual-level Internet usage frequency as an additional control variable to address potential omitted variable bias. The corresponding regression results are reported in column (3) of Table 4.

Finally, considering that China’s four major municipalities (Beijing, Shanghai, Tianjin, and Chongqing) have a larger migrant population and residents tend to enjoy more favorable policy support due to their special administrative status, we exclude samples from these municipalities and re-estimate the model. The results are presented in column (4) of Table 4.

Overall, across all robustness tests, the coefficient of the core explanatory variable—music industry digitalization (lndm)—remains significantly positive, confirming that the conclusion that digitalization of the music industry significantly enhances residents’ well-being is robust and reliable.

### 4.3. Mechanism analysis: the role of social participation, social trust, and mental health

After establishing the positive impact of music industry digitalization on residents’ happiness, this study further explores the underlying mechanisms through a mediation effect model. First, we empirically test whether social participation mediates the relationship between music industry digitalization and residents’ happiness. The corresponding regression results are presented in Table 5. As shown in Table 5, column (1) reports the baseline regression results without considering the mediating variable. Column (2) presents the regression results of music industry digitalization on social participation, where the coefficient for the core explanatory variable—music industry digitalization (lndm)—is significantly positive. This indicates that the development of the digital music industry significantly promotes residents’ social participation. Building on this, column (3) reports the results after introducing social participation (Gif) as an additional explanatory variable into the baseline model. The results show that the regression coefficient for social participation is significantly positive, suggesting that greater social participation significantly enhances residents’ happiness. Furthermore, compared to column (1), the magnitude and significance of the coefficient for lndm in column (3) are reduced, implying that part of the effect of music industry digitalization on happiness is mediated through social participation.

**Table 5:** Results of the Impact Mechanism Test for Social Participation.

	(1)	(2)	(3)
	hap	renq	hap
lndm	0.057^***^	0.191^***^	0.054^***^
	(3.17)	(8.52)	(2.99)
renq			0.017^***^
			(2.61)
age	−0.114^***^	0.092^***^	−0.116^***^
	(−15.84)	(10.27)	(−16.01)
age2	0.123^***^	−0.098^***^	0.125^***^
	(17.04)	(−10.84)	(17.21)
gender	0.018	−0.078^*^	0.019
	(0.55)	(−1.94)	(0.59)
edu	0.005	0.033^***^	0.004
	(1.13)	(5.98)	(1.01)
marital	0.869^***^	0.462^***^	0.862^***^
	(18.65)	(7.95)	(18.46)
hukou	−0.035	0.206^***^	−0.039
	(−0.90)	(4.20)	(−0.98)
job	−0.054	0.008	−0.054
	(−1.33)	(0.16)	(−1.33)
party	0.358^**^	0.166	0.355^**^
	(2.55)	(0.95)	(2.53)
ful	0.054^***^	0.214^***^	0.051^***^
	(6.21)	(19.67)	(5.74)
ec	0.108^***^	0.454^***^	0.101^***^
	(6.31)	(21.24)	(5.80)
Constant	7.898^***^	−1.564^***^	7.923^***^
	(30.54)	(−4.86)	(30.62)
Observations	15,967	15,971	15,967
R-squared	0.046	0.095	0.047
Region FE	YES	YES	YES

Notes: t-statistics in parentheses,

*** p < 0.01,

** p < 0.05,

* p < 0.1.

Combining the above findings, we conclude that the digitalization of the music industry promotes residents’ happiness by enhancing their level of social participation. Thus, **Hypothesis H1** is empirically supported.

Further, this study examines whether social trust serves as a mediating mechanism in the relationship between music industry digitalization and residents’ happiness. The corresponding regression results are presented in Table 6. As shown in Table 6, column (2) reports the regression of music industry digitalization (lndm) on social trust. The coefficient of lndm is significantly positive, indicating that the development of the digital music industry significantly enhances residents’ level of social trust.

**Table 6:** Results of the test of social trust influence mechanism.

	(1)	(2)	(3)
	hap	trust	hap
lndm	0.057^***^	0.110^***^	0.053^***^
	(3.17)	(5.48)	(2.93)
trust			0.039^***^
			(5.56)
age	−0.114^***^	−0.051^***^	−0.112^***^
	(−15.84)	(−6.33)	(−15.56)
age2	0.123^***^	0.045^***^	0.122^***^
	(17.04)	(5.53)	(16.80)
gender	0.018	0.537^***^	−0.003
	(0.55)	(14.83)	(−0.10)
edu	0.005	0.063^***^	0.003
	(1.13)	(12.74)	(0.57)
marital	0.869^***^	−0.023	0.870^***^
	(18.65)	(−0.44)	(18.69)
hukou	−0.035	−0.191^***^	−0.028
	(−0.90)	(−4.34)	(−0.71)
job	−0.054	0.029	−0.055
	(−1.33)	(0.64)	(−1.36)
party	0.358^**^	0.308^**^	0.346^**^
	(2.55)	(1.97)	(2.46)
ful	0.054^***^	0.001	0.054^***^
	(6.21)	(0.12)	(6.21)
ec	0.108^***^	0.120^***^	0.104^***^
	(6.31)	(6.27)	(6.04)
Constant	7.898^***^	2.002^***^	7.819^***^
	(30.54)	(6.94)	(30.22)
Observations	15,967	15,971	15,967
R-squared	0.046	0.072	0.048
Region FE	YES	YES	YES

Notes: t-statistics in parentheses,

*** p < 0.01,

** p < 0.05,

* p < 0.1.

As shown in Table 6, column (2) reports the regression of music industry digitalization (lndm) on social trust. The coefficient of lndm is significantly positive, indicating that the development of the digital music industry significantly enhances residents’ level of social trust. Building on this, column (3) presents the regression results after introducing social trust (Tru) as an additional explanatory variable. The results show that the coefficient of social trust is 0.039 and is statistically significant at the 1% level, suggesting that higher levels of social trust significantly improve residents’ happiness. Furthermore, compared to column (1), the absolute value and significance of the coefficient for music industry digitalization (lndm) in column (3) decrease after controlling for social trust, indicating that part of the effect of digitalization on happiness operates through the enhancement of social trust.

Based on these results, we conclude that the digitalization of the music industry promotes residents’ happiness by strengthening their social trust. Thus, **Hypothesis H2** is empirically supported.

Finally, this study investigates whether mental health acts as a mediating mechanism in the relationship between music industry digitalization and residents’ happiness. The corresponding empirical results are presented in Table 7. As shown in Table 7, column (2) reports the regression of music industry digitalization (lndm) on mental health. The coefficient of lndm is significantly positive, indicating that the digitalization of the music industry significantly improves residents’ mental health. Building on this, column (3) presents the regression results after including mental health (Mheal) as an additional explanatory variable. The results show that the coefficient of mental health is significantly positive, suggesting that better mental health has a significant positive effect on residents’ happiness. Moreover, compared to column (1), the absolute value and significance of the coefficient for music industry digitalization (lndm) decrease after controlling for mental health, indicating that part of the impact of digitalization on happiness operates through improvements in mental health.

**Table 7:** Results of the Mental Health Impact Mechanisms Test.

	(1)	(2)	(3)
	hap	mheal	hap
lndm	0.057^***^	0.001^***^	0.041^**^
	(3.17)	(2.71)	(2.42)
mheal			29.113^***^
			(42.89)
age	−0.114^***^	−0.001^***^	−0.097^***^
	(−15.84)	(−7.64)	(−14.11)
age2	0.123^***^	0.001^***^	0.103^***^
	(17.04)	(8.96)	(14.92)
gender	0.018	0.003^***^	−0.084^***^
	(0.55)	(9.75)	(−2.72)
edu	0.005	0.000^***^	−0.009^**^
	(1.13)	(10.00)	(−2.19)
marital	0.869^***^	0.006^***^	0.684^***^
	(18.65)	(12.39)	(15.43)
hukou	−0.035	−0.002^***^	0.010
	(−0.90)	(−3.52)	(0.26)
job	−0.054	−0.001	−0.034
	(−1.33)	(−1.49)	(−0.89)
party	0.358^**^	0.002	0.305^**^
	(2.55)	(1.17)	(2.29)
ful	0.054^***^	0.000^***^	0.045^***^
	(6.21)	(3.34)	(5.43)
ec	0.108^***^	0.001^***^	0.067^***^
	(6.31)	(7.52)	(4.11)
Constant	7.898^***^	0.065^***^	5.993^***^
	(30.54)	(22.89)	(24.08)
Observations	15,967	15,971	15,967
R-squared	0.046	0.051	0.145
Region FE	YES	YES	YES

Notes: t-statistics in parentheses,

*** p < 0.01,

** p < 0.05,

* p < 0.1.

Based on these findings, we conclude that the digitalization of the music industry promotes residents’ happiness by enhancing their mental health. Therefore, **Hypothesis H3** is empirically supported

### 4.4. Heterogeneity analysis: differential impacts across internet usage, education, and economic Status

After empirically examining the overall relationship between music industry digitalization and residents’ happiness, as well as its underlying mechanisms, this study further investigates the heterogeneity in the effects of digitalization across different groups. Specifically, we explore whether the impact of music industry digitalization on residents’ well-being varies according to Internet usage frequency, education level, and economic status.

First, we use the CFPS survey question on “the length of time spent on the Internet via mobile devices” as a proxy for respondents’ Internet access frequency. Using the median value as the threshold, the sample is divided into high Internet access and low Internet access groups, and group regressions are conducted. The corresponding results are presented in Table 8. As shown in Table 8, for the group with low Internet access, the coefficient of music industry digitalization (lndm) is 0.112 and statistically significant at the 1% level. In contrast, for the group with high Internet access, the coefficient of lndm is 0.019 and fails to reach statistical significance. These results suggest that the positive impact of music industry digitalization on residents’ happiness is more pronounced among individuals with lower levels of Internet access compared to those with higher levels.

**Table 8:** Heterogeneity in the degree of internet access for the impact of music industry digitisation on residents’ happiness.

	(1)	(2)
	Nonhigh_int	high_int
	hap	hap
lndm	0.112^***^	0.019
	(3.90)	(0.84)
age	−0.102^***^	−0.134^***^
	(−8.47)	(−12.59)
age2	0.111^***^	0.144^***^
	(9.85)	(12.35)
gender	−0.040	0.099^**^
	(−0.81)	(2.36)
edu	−0.001	0.021^***^
	(−0.22)	(3.26)
marital	0.709^***^	1.018^***^
	(9.48)	(17.41)
hukou	−0.054	−0.028
	(−0.83)	(−0.58)
job	−0.154^**^	0.030
	(−2.51)	(0.56)
party	0.455	0.296^**^
	(1.44)	(2.04)
ful	0.054^***^	0.049^***^
	(4.26)	(4.02)
ec	0.129^***^	0.082^***^
	(5.29)	(3.29)
Constant	7.794^***^	8.186^***^
	(18.69)	(22.34)
Observations	8,062	7,905
R-squared	0.043	0.058
Region FE	YES	YES

Notes: t-statistics in parentheses,

*** p < 0.01,

** p < 0.05,

* p < 0.1.

Further, this study uses the “years of education completed by individual respondents” from the CFPS questionnaire as a proxy for respondents’ education levels. Using the median value as a cutoff point, the sample is divided into two groups: low education level and high education level. Group regressions are then conducted to explore the heterogeneity of the impact of music industry digitalization on residents’ well-being across education levels. The corresponding results are presented in Table 9.

**Table 9:** Heterogeneity of educational attainment in the impact of music industry digitisation on residents’ well-being.

	(1)	(2)
	low_edu	high_edu
	hap	hap
lndm	0.122^***^	−0.024
	(4.78)	(−1.01)
age	−0.102^***^	−0.118^**^*
	(−10.24)	(−10.33)
age2	0.113^***^	0.125^***^
	(11.60)	(10.27)
gender	−0.002	0.087^*^
	(−0.04)	(1.91)
edu	−0.002	0.032^**^
	(−0.27)	(2.50)
marital	0.910^***^	0.841^***^
	(13.89)	(13.18)
hukou	−0.057	−0.028
	(−0.96)	(−0.56)
job	−0.069	−0.001
	(−1.25)	(−0.01)
party	0.408	0.280^**^
	(1.14)	(2.11)
ful	0.059^***^	0.050^***^
	(5.23)	(3.61)
ec	0.104^***^	0.107^***^
	(4.69)	(3.94)
Constant	7.624^***^	7.657^***^
	(21.69)	(18.15)
Observations	10,024	5,943
R-squared	0.049	0.047
Region FE	YES	YES

Notes: t-statistics in parentheses,

*** p < 0.01,

** p < 0.05,

* p < 0.1.

As shown in Table 9, for the group with lower education levels, the coefficient of music industry digitalization (lndm) is 0.122 and statistically significant at the 1% level. In contrast, for the group with higher education levels, the coefficient of lndm is −0.024 and not statistically significant. These findings indicate significant heterogeneity: the positive impact of music industry digitalization on residents’ well-being is more pronounced among those with lower education levels, while the effect is negligible among those with higher education levels.

Finally, this study uses the CFPS survey question “your income position in the local area” as a proxy for respondents’ economic status. Responses to this question range from 1 to 5, with higher values indicating that respondents perceive themselves as having a higher economic status relative to their local peers. Accordingly, we classify respondents with a value greater than 3 as having high local economic status and the rest as having low local economic status. Group regressions are conducted to explore the heterogeneity of the impact of music industry digitalization on residents’ well-being across different economic status groups. The corresponding results are presented in Table 10

**Table 10:** Results of economic status heterogeneity in the impact of music industry digitisation on residents’ happiness.

	(1)	(2)
	low_ecol	high_ecol
	hap	hap
lndm	0.073^***^	0.036
	(3.62)	(0.95)
age	−0.111^***^	−0.081^***^
	(−13.35)	(−5.75)
age2	0.117^***^	0.089^***^
	(13.82)	(6.41)
gender	0.010	0.044
	(0.28)	(0.65)
edu	0.019^***^	−0.002
	(3.76)	(−0.24)
marital	0.937^***^	0.513^***^
	(18.09)	(5.18)
hukou	−0.048	−0.067
	(−1.12)	(−0.77)
job	−0.021	−0.182^**^
	(−0.45)	(−2.28)
party	0.353^**^	0.061
	(2.21)	(0.22)
ful	0.045^***^	0.049^***^
	(4.55)	(2.90)
ec	0.095^***^	0.088^***^
	(4.85)	(2.63)
Constant	7.652^***^	8.619^***^
	(25.83)	(17.08)
Observations	12,302	3,665
R-squared	0.050	0.033
Region FE	YES	YES

Notes: t-statistics in parentheses,

*** p < 0.01,

** p < 0.05,

* p < 0.1.

As shown in Table 10, for the group with lower economic status, the coefficient of music industry digitalization (lndm) is 0.073 and statistically significant. In contrast, for the group with higher economic status, although the coefficient of lndm is positive, it is not statistically significant. These results suggest that the positive impact of music industry digitalization on residents’ happiness is relatively stronger among individuals with lower economic status compared to those with higher economic status.

## 5. Discussion and conclusion

### 5.1. Discussion of results

Based on the Stimulus-Organism-Response theoretical framework, this study deeply explores the impact of digitalisation in the music industry on residents’ subjective well-being, as well as its underlying mechanisms. The empirical findings validate all research hypotheses proposed in this study.

First, the baseline result demonstrates that digitalisation of the music industry significantly enhances residents’ subjective well-being, aligning with the findings of previous studies, such as North & Hargreaves [2] and Morinville & Miranda [23], which have similarly concluded that music consumption positively influences individual well-being. However, this study extends the existing literature—which primarily focuses on the direct impact of music consumption—by thoroughly delineating a comprehensive SOR-based pathway. Specifically, it reveals how digitalisation of the music industry promotes residents’ happiness through three distinct mediating mechanisms: social participation, social trust, and mental health, thereby broadening the theoretical interpretation of the social effects of digital cultural consumption.

Second, regarding the mechanism of social participation, the empirical results confirm hypothesis H1, which states that the development of digital music enhances residents’ happiness by increasing their level of social participation. This finding resonates with prior research by Oestreicher-Singer & Zalmanson [25], who indicated that digital platforms significantly enhance users’ social engagement and social capital. By further leveraging social capital theory, this study illustrates how digital music platforms, through built-in social sharing features, interactive comment sections, and virtual community creation, effectively elevate users’ sense of social belonging and participation, thereby significantly enhancing subjective well-being. The identification and validation of this mechanism enrich the application of social capital theory in the context of digital music consumption.

Third, concerning the social trust mechanism, this study similarly finds that digitalisation of the music industry significantly improves residents’ social trust (H2). This result corroborates the findings of Rothaermel & Sugiyama [29], who argued that interactions within virtual communities foster greater social trust among individuals. It is suggested that music platforms construct virtual social spaces through emotional resonance and collective identity, effectively reducing psychological barriers and risk perception between strangers, consequently enhancing overall social trust and subjective well-being. Thus, this study refines the application of social trust theory in digital music consumption scenarios, expanding its explanatory power.

Additionally, regarding the mental health mechanism, empirical results substantiate hypothesis H3, indicating that digitalisation in the music industry significantly improves residents’ mental health, thereby enhancing their subjective well-being. This finding aligns with the conclusions of Terry & Karageorghis [30] and Block & Wong [31], who posited positive mental health effects from music consumption. Using emotion regulation theory, this study further elaborates how digital music, through diversified and personalized emotional regulation tools and content, enables residents to effectively regulate and express emotions anytime and anywhere, alleviating daily anxiety, depression, and stress, and thus markedly improving mental health and happiness. This study uniquely highlights the critical roles of immediacy and customization in digital music for enhancing mental health, enriching the theoretical application of mental health within digital cultural consumption contexts.

Finally, heterogeneity analysis across dimensions of internet usage frequency, educational attainment, and economic status reveals that the positive impact of digitalisation in the music industry on happiness is more pronounced among groups with lower internet access frequency, lower educational levels, and lower economic status. This finding supports the digital divide theory [37]. It primarily arises because digital music significantly reduces the cultural consumption and emotional interaction barriers faced by disadvantaged groups, offering them accessible entertainment content and channels for social engagement, thus alleviating social isolation and psychological stress resulting from limited social resources. This redistribution and compensation of cultural and emotional resources provides essential support for enhancing happiness among socially disadvantaged groups, underscoring digital music’s significant role in promoting social equity and psychological well-being.

In summary, by integrating and applying the SOR theory, this study thoroughly analyses the specific mechanisms by which digitalisation in the music industry enhances residents’ subjective well-being. It clearly delineates the relationship between the study’s findings and existing theoretical frameworks and literature, thereby further enriching the theoretical system concerning digital cultural consumption and social well-being, and offering significant theoretical innovation and practical implications.

### 5.2. Research conclusions

In the wave of advanced digital technologies sweeping across various industries, the rapid development of the digital music industry has not only fundamentally changed the modes of music production, dissemination, and consumption but has also gradually permeated people’s daily lives, becoming an important component of residents’ entertainment, leisure, and cultural activities. It has exerted a significant influence on the mental health of populations worldwide. Based on micro-level data from the 2022 China Family Panel Studies (CFPS), this study empirically examines the role of music industry digitalization on residents’ well-being and its underlying mechanisms at the individual level, providing novel micro-level evidence for understanding the impact of digital music on individual well-being from a new perspective.

The main findings of this study are as follows:

(1) Baseline regression results indicate that the digitalization of the music industry significantly enhances residents’ well-being.
(2) Mechanism analysis results reveal that digital music improves residents’ well-being by enhancing social participation, strengthening social trust, and promoting mental health.
(3) Heterogeneity analysis results show that the positive effect of music industry digitalization on well-being is more pronounced among individuals with lower Internet usage frequency, lower educational attainment, and lower economic status, compared to their higher-frequency, higher-education, and higher-income counterparts.

Based on these main findings, this study proposes the following policy recommendations:

First, fully leverage the role of online digital music platforms in driving the development of digital music. The government should guide platforms through supportive policies to not only strengthen copyright protection but also actively enhance social interaction functions and personalized recommendation systems. This would further amplify the platforms’ social attributes, positioning them as important spaces for emotional exchange and social interaction among users.

Second, promote the social attributes of digital music to enhance cultural vitality. Governments could encourage digital music creators, particularly independent artists, by establishing dedicated funding programs and reward mechanisms for creative innovation. At the same time, support should be provided for digital music platforms to host online music events and creator competitions, fostering cultural and emotional exchanges within digital communities. These initiatives would not only strengthen the social identity of music creators but also promote the diversification and flourishing of musical culture, enriching society with more artistic and cultural products.

Third, strengthen ethical regulation of digital music platforms and enhance user privacy protection. With the increasing social and interactive functions of digital music platforms, issues related to user data and privacy have become a growing public concern. The government should intensify the regulation of digital music platforms to ensure strict compliance with data privacy and user security management standards in all social interactions. Platforms should also be encouraged to publish transparent privacy policies and undergo regular compliance reviews, ensuring that users enjoy strong privacy protections and information security while participating in social interactions. This, in turn, would help safeguard users’ perceptions of social trust in the digital environment.

### 5.3. Research limitations

Although this study utilizes official song list data from major Chinese digital music platforms and micro-level survey data to explore a large sample, several limitations remain. First, digital music today encompasses not only online streaming music but also emerging formats such as online concerts and virtual performances. Due to limitations in data availability and usability, this study focuses solely on online music data and does not incorporate other forms of digital music products. Future research could expand the scope by collecting larger-scale data, covering more diverse formats of digital music consumption to provide a more comprehensive analysis. Second, the rapid development of the digital music industry has led to the emergence of various music genres, formats, and audience labels. However, this study treats digital music as a homogeneous category and does not differentiate the specific effects of different types of music on residents’ well-being. Future studies could conduct more detailed heterogeneity analyses by collecting and classifying digital music data according to genre, format, or target audience characteristics, thus offering a more nuanced understanding of how different forms of digital music influence individual well-being.
